# Typing of Lymphogranuloma Venereum *Chlamydia trachomatis* Strains

**DOI:** 10.3201/eid1611.100379

**Published:** 2010-11

**Authors:** Linus Christerson, Henry J.C. de Vries, Bertille de Barbeyrac, Charlotte A. Gaydos, Birgit Henrich, Steen Hoffmann, Julius Schachter, Johannes Thorvaldsen, Martí Vall-Mayans, Markus Klint, Björn Herrmann, Servaas A. Morré

**Affiliations:** Author affiliations: Uppsala University, Uppsala, Sweden (L. Christerson, M. Klint, B. Herrmann);; Municipal Health Service Amsterdam, Amsterdam, the Netherlands (H.J.C. de Vries);; University of Amsterdam, Amsterdam (H.J.C. de Vries);; National Institute for Public Health and the Environment, Bilthoven, the Netherlands (H.J.C. de Vries);; Université Victor Segalen Bordeaux 2, Bordeaux, France (B. de Barbeyrac);; Johns Hopkins University, Baltimore, Maryland, USA (C.A. Gaydos);; Heinrich-Heine-University, Duesseldorf, Germany (B. Henrich);; Statens Serum Institut, Copenhagen, Denmark (S. Hoffman);; University of California, San Francisco, California, USA (J. Schachter);; Oslo University Hospital, Oslo, Norway (J. Thorvaldsen);; Catalan Health Institute, Barcelona, Spain (M. Vall-Mayans);; VU University Medical Center, Amsterdam (S.A. Morré)

**Keywords:** Chlamydia trachomatis, lymphogranuloma venereum, outer membrane protein A, multilocus sequence typing, men who have sex with men, Europe, United States, bacteria, dispatch

## Abstract

We analyzed by multilocus sequence typing 77 lymphogranuloma venereum *Chlamydia trachomatis* strains from men who have sex with men in Europe and the United States. Specimens from an outbreak in 2003 in Europe were monoclonal. In contrast, several strains were in the United States in the 1980s, including a variant from Europe.

Lymphogranuloma venereum (LGV) is a sexually transmitted disease caused by *Chlamydia trachomatis* serovars L1, L2, and L3. This disease is endemic to parts of Africa, Latin America, and Asia but only occurred sporadically in Europe before 2003, at which time an outbreak of LGV occurred in the Netherlands among men who have sex with men (MSM) (*1*). Since then, a large number of LGV cases have been reported among MSM in Europe, North America, and Australia. Although the inguinal form (formation of buboes) is more common in heterosexual LGV patients, in the current epidemic among MSM, anal infections have been diagnosed in most LGV cases.

Sequencing the highly variable outer membrane protein A (*ompA*) gene identified a new genetic variant designated L2b, which subsequently has been identified in nearly all recent LGV cases in MSM that have been investigated (*2*). This variant was also found in isolates obtained from MSM in San Francisco, California, USA, in the early 1980s (*3*). However, differences in other regions of the *C. trachomatis* genome are possible and these regions should be investigated.

Multilocus sequence typing (MLST) is a genotyping method based on amplification and sequencing of several genetic regions. Recently, 3 MLST systems for genotyping of *Chlamydiacae* bacteria have been reported. Two are based on housekeeping genes, and their resolution is comparable to that of *ompA* (*4*,*5*), which gives these 2 systems limited usefulness in *C. trachomatis* strain discrimination and outbreak investigations. A third MLST system was reported by Klint et al. (*6*) for short-term epidemiologic analysis of *C. trachomatis* strains. This system showed a 3-fold higher resolution than conventional *ompA* genotyping when applied to serovars A–K (*6*,*7*), which cause ocular trachoma and genital chlamydia infections. Recent evaluation of typing schemes confirms the considerable discriminatory potential of our system and recommends it for typing of closely related clinical strains (*8*). This system includes 5 highly variable gene regions; 2 of them (penicillin-binding protein and histone H1–like protein [*hctB*]), are subjected to selection pressure, which facilitates analysis of epidemiologic changes over limited periods.

Our objective was to deduce the nature and origin of the LGV outbreak among MSM in Europe. We used the MLST system of Klint et al. (*6*) to investigate genetic variation in LGV strains. Strains were obtained from MSM from contemporary Europe and the United States and from MSM in San Francisco during the 1980s.

## The Study

All LGV specimens were obtained from MSM attending outpatient clinics. Twenty-two specimens were obtained in San Francisco during 1979–1985, and 5 specimens were obtained in the Baltimore, MD–Washington, DC, USA, area during 2007–2009. Fifty specimens were obtained from patients in Europe (Denmark [n = 7], France [n = 15], Germany [n = 1], the Netherlands [n = 9], Norway [n = 2], Spain [n = 7], and Sweden [n = 9]) during 2004–2008. DNA purification, PCR amplification of the *ompA* gene and the 5 specific highly variable gene regions (by using high-fidelity polymerase), DNA sequencing, and analysis of data (mean 5,972 nt/specimen, primers excluded) were performed as described (*6*,*9*). All novel mutations were reamplified and resequenced to confirm their authenticity.

All except 1 of the 50 specimens from Europe had an *ompA* genotype identical to the L2b reference strain L2b/UCH-1/proctitis (AM884177.1). The nonidentical specimen came from Spain and had a previously unpublished single C→T point mutation in variable segment 2 at position 517 when compared with L2b/UCH-1/proctitis. This sequence of this specimen has been deposited in GenBank (accession no. GQ413955). All 50 specimens from Europe shared an identical MLST genotype (Table). The 5 specimens from the contemporary United States had the same *ompA* and MLST genotype as specimens from Europe (Table).

**Table:** Genetic profiles of lymphogranuloma venereum specimens, Europe and United States*

Location	Sample years	No. specimens	MLST profile					*ompA*
			*hctB*	CT058	CT144	CT172	*pbpB*	
Europe†	2004–2009	49	27	13	17	13	29	28‡
Europe§	2004–2009	1	27	13	17	13	29	39¶
USA#	2007–2009	5	27	13	17	13	29	28‡
USA**	1979–1985	3	27	13	17	13	29	28‡
USA**	1979–1985	6	44††	13	17	13	29	28‡
USA**	1979–1985	7	18	13	23	13	29	40‡‡
USA**	1979–1985	5	18	13	19	6	28	22§§
USA**	1979–1985	1	18	37	19	6	28	22§§

*MLST, multilocus sequence typing; *hctB*, histone H1–like protein; *pbpB*, penicillin-binding protein; *ompA*, outer membrane protein A. Values are arbitrary designations referring to allele variants in our *Chlamydia trachomatis* MLST database (http://mlstdb.bmc.uu.se/). All MLST variants differ within regions with <5 point mutations unless otherwise indicated. †Denmark (n = 7), France (n = 15), Germany (n = 1), the Netherlands (n = 9), Norway (n = 2), Spain (n = 6), and Sweden (n = 9). ‡*ompA* variant 28 is identical to the reference strain L2b/UCH-1/proctitis (AM884177.1). §Spain. ¶*ompA* variant 39 contains a single point mutation compared with L2b/UCH-1/proctitis and has been deposited in GenBank under accession no. GQ413955. #Baltimore, Maryland–Washington, DC. **San Francisco, California. ††*hctB* variant 44 contains a novel 108-nt deletion and is unique in the MLST database. ‡‡*ompA* variant 40 contains 9 point mutations compared with reference strain L1/440 (DQ064294.1) and has been deposited in GenBank under accession no. GQ413956. §§*ompA* variant 22 is identical to reference strain L2/434/Bu (AM884176.1).

The 22 specimens from San Francisco were separated into 3 *omp*A genotypes and 5 MLST genotypes (Table). Six specimens had an *ompA* genotype identical to the L2 reference strain L2/434/Bu, and 7 had a novel genotype that differed from the L1 reference strain L1/440 by 9 point mutations. The remaining 9 specimens had an *ompA* genotype identical those of specimens from Europe and L2b reference strain L2b/UCH-1/proctitis. Three of these 9 specimens had MLST genotypes of strains from Europe, and the other 6 had a novel 108-nt deletion in the *hctB* gene region. This *hctB* variant has not been found in other specimens of *C. trachomatis* serovars A–L3 and is unique in the MLST database (http://mlstdb.bmc.uu.se/), which contains genetic profiles for 496 specimens (February 2010).

## Conclusions

We aimed to deduce the nature and origin of the LGV outbreak among MSM in Europe. Because of the conserved nature of *Chlamydia* spp. genomes, high-resolution genotyping, rather than following evolutionary changes over long periods as with conventional MLST systems, was essential for investigating this outbreak. We found that the high-resolution MLST system described by Klint et al. (*6*) was most suitable for the current study.

LGV specimens from MSM in Europe in this study were monoclonal for *ompA* and the 5 highly variable regions. This finding suggests a single source of origin for the LGV outbreak among MSM in Europe. One specimen in this study contained a novel, single, point mutation in *ompA*. In 373 contemporary LGV cases in MSM in the United Kingdom, the L2b *ompA* variant comprised >90% of the specimens, and the remaining 35 specimens belonged to 4 novel sequence variants, each differing from the L2b variant by a single point mutation (*10*). These single point mutations might have occurred recently, and their presence is less likely to indicate a separate source of origin.

LGV specimens from MSM in the 1980s in San Francisco showed genetic variation in *ompA* and the 5 highly variable gene regions. MLST analysis showed that 6 of the 9 specimens from San Francisco that had the L2b *ompA* variant were genetically different from the strain in Europe; the remaining 3 specimens had an MLST profile identical to that of the variant from Europe. Genetic variation among LGV specimens from San Francisco supports the idea that LGV is endemic in MSM in the United States, a finding that has been reported (*11*).

In contrast, the epidemiologic pattern and genetic characterization of LGV in several countries in Europe indicate that the L2b type has disseminated across Europe in recent years. In Sweden, 3 LGV cases that produced clinical signs were detected in 2004 and 2005 (*12*). In a survey of 81% of patients with *C. trachomatis* infection detected among high risk MSM in Stockholm, no additional LGV cases were detected (*12*), and a total of 15 LGV cases were detected in 2007. If one considers the highly internationalized network of sexual contacts among MSM (*13*), the L2b variant may have been imported to Europe from the United States. A limitation of our study is that it does not include old LGV strains from Europe. However, after widespread investigations, we believe that no such strains are available.

Our study shows that the MLST system of Klint et al. (*6*) is suitable for epidemiologic analysis of *C. trachomatis* transmission, as indicated by our ability to differentiate the L2b *ompA* variant found in San Francisco into 2 strains. Investigation of additional LGV specimens from the United States and regions to which LGV is endemic with the MLST system could help determine a more detailed epidemiologic picture of the LGV outbreak in 2003 among MSM.
